# Prenatal PFAS and psychosocial stress exposures in relation to fetal growth in two pregnancy cohorts: Applying environmental mixture methods to chemical and non-chemical stressors

**DOI:** 10.1016/j.envint.2022.107238

**Published:** 2022-04-09

**Authors:** Stephanie M. Eick, Elizabeth A. Enright, Amy M. Padula, Max Aung, Sarah D. Geiger, Lara Cushing, Jessica Trowbridge, Alexander P. Keil, Hyoung Gee Baek, Sabrina Smith, June-Soo Park, Erin DeMicco, Susan L. Schantz, Tracey J. Woodruff, Rachel Morello-Frosch

**Affiliations:** a Gangarosa Department of Environmental Health, Rollins School of Public Health, Emory University, Atlanta, GA, USA; b Department of Psychology, St. Mary’s College of Maryland, St. Mary’s City, MD, USA; c Program on Reproductive Health and the Environment, Department of Obstetrics, Gynecology and Reproductive Sciences, University of California, San Francisco, San Francisco, CA, USA; d Division of Environmental Health, Department of Population and Public Health Sciences, Keck School of Medicine, University of Southern California; e Beckman Institute for Advanced Science and Technology, University of Illinois at Urbana-Champaign, Champaign, IL USA; f Department of Kinesiology and Community Health, University of Illinois at Urbana-Champaign, Champaign, IL, USA; g Department of Environmental Health Sciences, Fielding School of Public Health, University of California, Los Angeles, CA, USA; h Department of Epidemiology, University of North Carolina at Chapel Hill, Chapel Hill, NC, USA; i Environmental Chemistry Laboratory, Department of Toxic Substances Control, California Environmental Protection Agency, Berkeley, CA, USA; j Department of Comparative Biosciences, University of Illinois at Urbana-Champaign, Champaign, IL, USA; k Department of Environmental Science, Policy and Management and School of Public Health, University of California, Berkeley, CA, USA

**Keywords:** Per- and poly-fluoroalkyl substances, Stress, Pregnancy, Mixtures

## Abstract

**Background:**

Prenatal exposure to individual per- and poly-fluoroalkyl substances (PFAS) and psychosocial stressors have been associated with reductions in fetal growth. Studies suggest cumulative or joint effects of chemical and non-chemical stressors on fetal growth. However, few studies have examined PFAS and non-chemical stressors together as a mixture, which better reflects real life exposure patterns. We examined joint associations between PFAS, perceived stress, and depression, and fetal growth using two approaches developed for exposure mixtures.

**Methods:**

Pregnant participants were enrolled in the Chemicals in Our Bodies cohort and Illinois Kids Development Study, which together make up the ECHO.CA.IL cohort. Seven PFAS were previously measured in 2nd trimester maternal serum samples and were natural log transformed for analyses. Perceived stress and depression were assessed using self-reported validated questionnaires, which were converted to t-scores using validated methods. Quantile g-computation and Bayesian kernel machine regression (BKMR) were used to assess joint associations between PFAS, perceived stress and depression t-scores and birthweight z-scores (N = 876).

**Results:**

Individual PFAS, depression and perceived stress t-scores were negatively correlated with birthweight z-scores. Using quantile g-computation, a simultaneous one quartile increase in all PFAS, perceived stress and depression t-scores was associated with a slight reduction in birthweight z-scores (mean change per quartile increase = −0.09, 95% confidence interval = −0.21, 0.03). BKMR similarly indicated that cumulative PFAS and stress t-scores were modestly associated with lower birthweight z-scores. Across both methods, the joint association appeared to be distributed across multiple exposures rather than due to a single exposure.

**Conclusions:**

Our study is one of the first to examine the joint effects of chemical and non-chemical stressors on fetal growth using mixture methods. We found that PFAS, perceived stress, and depression in combination were modestly associated were lower birthweight z-scores, which supports prior studies indicating that chemical and non-chemical stressors are jointly associated with adverse health outcomes.

## 1. Introduction

Per- and poly-fluoroalkyl substances (PFAS) are a group of synthetic chemicals that are or were widely detected and persistent in the environment and are used in a variety of consumer products, including non-stick cookware and paper food packing containers, due to their oil and water repellant properties (Sunderland et al., 2019). Representative studies of the US population have shown that 100% of individuals have detectable levels of the PFAS chemicals, perfluorooctanoic acid (PFOA) and perfluorooctane sulfonic acid (PFOS) in their blood serum (Calafat et al., 2007; Andrews and Naidenko, 2020). This is particularly problematic for pregnant people, as PFOA and PFOS are detectable in the placenta and in the umbilical cord blood of newborns, oftentimes at equal or greater concentrations in the fetus compared to the mother (Morello-Frosch et al., 2016; Kang et al., 2021; Panagopoulos Abrahamsson et al., 2021). A recent systematic review concluded that PFOA and PFOS were associated with an increased risk of delivering preterm (Deji et al., 2021). However, systematic reviews examining the relationship between PFAS, birthweight, and small and large for gestational age are inconsistent (Steenland et al., 2018; Dzierlenga et al., 2020; Gao et al., 2021). Psychosocial stressors and responses to stress, including depression and anxiety, are also highly prevalent during pregnancy and may be linked to adverse birth outcomes. For example, maternal experiences of stressful life events and poor perceived neighborhood quality have been shown to be associated with increased preterm birth risk (Dole et al., 2003). Experiencing food insecurity in combination with stressful life events and unplanned pregnancy also adversely influences fetal growth (Goin et al., 2021). Pregnant people who experience stressors such as job strain, discrimination, and stressful life events often report higher levels of perceived stress and depression (Eick et al., 2020), indicating that perceived stress and depression occur as a response to psychosocial stressors.

A growing number of studies suggest that joint exposure to both chemical and non-chemical stressors is associated with synergistic, reduction in gestational age at birth and fetal growth (Padula et al., 2020; Vesterinen et al., 2017; Barrett and Padula, 2019). For example, the relationship between phenols, phthalates, and preterm birth was stronger among those who experienced stressful life events among pregnancy cohorts in the US and Puerto Rico (Aker et al., 2020; Ferguson et al., 2019). Relative to those without high levels of psychosocial stress, high levels of psychosocial stress amplified the association between manganese and preterm birth (Ashrap et al., 2021). PFAS and non-chemical stressors may also be acting on the same biologic pathways. We recently showed that the relationship between perfluorononanoic acid (PFNA) and corticotropin-releasing hormone (CRH, a biomarker of stress) was stronger among those who experienced stressful life events, depression, food insecurity and financial strain (Eick et al., 2022), highlighting a potential biologic mechanism linking chemical and non-chemical stressors to preterm birth (Latendresse, 2009), This is supported by findings from toxicological studies, showing that adverse outcomes associated with chemical exposures (lead, concentrated ambient particles, diesel exhaust particles) are greater in magnitude among mice and rats with stress (Clougherty et al., 2014). Further, psychosocial stress activates the hypothalamic–pituitary–adrenal (HPA) axis, leading to changes in endocrine function and heighted susceptibility to chemicals (Clougherty et al., 2014).

For understanding the synergistic and additive effects of social stressors and environmental exposures, prior studies have been largely limited by their focus on independent associations of single pollutants or social stressors. Inference from single pollutant models is subject to confounding by important, correlated co-exposures. Studies that have examined joint exposure to both chemical and non-chemical stressors have largely done so using stratified analysis, which examines interaction between an environmental mixture and only one social stressor at a time. This is problematic, because numerous stressors can occur simultaneously, many of which reflect socioeconomic strata. Recently, statistical methods have been developed to analyze the joint effects of chemical mixtures, which controls confounding by correlated co-exposures and reflects the reality that exposure to one chemical often implies exposure to others. However, these methods have not routinely incorporated non-chemical stressors, which is warranted due to the correlation between environmental and social stressors, as well as evidence of synergism between them (Padula et al., 2020; Barrett and Padula, 2019).

In the present analysis, we adapted two mixture methods, quantile g-computation and Bayesian kernel machine regression (BKMR) to examine the cumulative association between PFAS and non-chemical stressors on fetal growth, as indicated by birthweight for gestational age z-scores. We hypothesized that the joint effects of multiple PFAS and non-chemical stressors would be stronger than the what was observed for a single PFAS or a single stressor alone. We utilized the Chemicals in Our Bodies (CIOB) cohort and the Illinois Kids Development Study (IKIDS), which together make up the ECHO.CA.IL cohort. Our study population included a racially and demographically diverse sample of pregnant people in San Francisco, CA and Urbana-Champaign, IL with a wide range of exposure to psychosocial stress and background range exposure to PFAS.

## 2. Methods

### 2.1. Study population

The ECHO.CA.IL study population consists of two longitudinal pregnancy cohorts launched separately in Urbana-Champaign, IL (IKIDS) and San Francisco, CA (CIOB). These two cohorts merged in 2016 as part of the National Institutes of Health (NIH) Environmental influences on Child Health Outcomes (ECHO) program; recruitment is ongoing. The goal of the ECHO.CA.IL cohort is to examine the individual and cumulative effects of chemical and non-chemical stressors on birth outcomes and neurodevelopment in infancy and early childhood. Detailed information about these cohorts is provided elsewhere (Eick et al., 2021). Briefly, CIOB participants were recruited during the second trimester and were eligible for inclusion if they were at least 18 years of age, spoke English or Spanish as their primary language, and were not pregnant with multiples. Inclusion criteria for IKIDS was as follows: less than 15 weeks gestation, between 18 and 40 years of age, not pregnant with multiples, spoke English as a primary language, low-risk pregnancy, not already enrolled in the study with another child, and resided within a 30-minute drive of Champaign, IL. All participants provided written, informed consent prior to participating in the study. The Institutional Review Boards at the University of California, San Francisco (10–00861) and Berkeley (2010–05-04), and University of Illinois, Urbana-Champaign (09498) approved CIOB and IKIDS, respectively.

### 2.2. Per- and poly-fluoroalkyl substances (PFAS)

Serum samples were obtained from CIOB participants during the second trimester (range 12–28 weeks) and from IKIDS participants between 16- and 20- weeks’ gestation. Prior to analysis, serum samples were stored at −80 °C. The Environmental Chemical Laboratory at the California Department of Toxic Substances Control (DTSC) quantified 12 PFAS in both cohorts: PFOA, PFOS, PFNA, perfluoro butane sulfonate (PFBS), perfluorohexanesulphonic acid (PFHxS), perfluoroheptanoic acid (PFHpA), perfluorodecanoic acid (PFDeA), perfluoroundecanoic acid (PFUdA), perfluorododecanoic acid (PFDoA), perfluorooctane sulfonamide (PFOSA), methyl-perfluorooctane sulfonamide acetic acid (Me- PFOSA-AcOH), ethyl-perfluorooctane sulfonamide acetic acid (Et-PFOSA-AcOH). Briefly, PFAS were quantified by injection into an automated on-line solid phase extraction instrument or module coupled to liquid chromatography and tandem mass spectrometry. Additional information regarding this method is provided in detail elsewhere.(Eick et al., 2020). We focused our analysis on those PFAS with ≥ 70% detection in the combined cohort (Clarity et al., 2021; Trowbridge et al., 2020), which included PFOA, PFOS, PFNA, PFHxS, PFDeA, PFUdA, and Me-PFOSA-AcOH. MDL values are provided in Table S1. For values below the method detection limit (MDL), we assigned the machine read value if it was available, as has been done previously (Eick et al., 2022; Eick et al., 2020). If there was no machine read value, the concentration was replaced with the $\text{MDL}/\sqrt{2}$, as is recommended when data are not highly skewed (Hornung and Reed, 1990). All PFAS were natural log transformed for analysis.

### 2.3. Responses to psychosocial stress

Perceived stress and depression were included as indicators of psychosocial stress response. Previously in the CIOB study population, we observed that perceived stress and depression were downstream consequences of stressful life events, discrimination, food insecurity, and job strain (Eick et al., 2020). Thus, we conceptualized perceived stress and depression as responses to exposure to multiple psychosocial stressors.

Perceived stress and depression were assessed during the second trimester in CIOB and between 10 and 14 weeks gestation in IKIDS. We measured perceived stress using the Perceived Stress Scale (PSS)-4 in CIOB and the PSS-10 in IKIDS (Cohen, 1988; Cohen et al., 1983). PSS-4 and PSS-10 scores were harmonized by converting PSS scores to t-scores using the NIH toolbox method, based on item response theory (IRT) and is normalized to the adult US general population. This method is described in detail elsewhere (Kupst et al., 2015). Depression was assessed using the Center for Epidemiologic Studies-Depression (CES-D) scale in CIOB and the Edinburg Postnatal Depression Scale (EPDS) in IKIDS (Radloff, 1977; Cox et al., 1987). Depression scores were similarly converted to t-scores by using crosswalk tables to convert non-PROMIS measures to scores on the PROMIS Emotional Distress—Depression: Short Form T-score metric (Blackwell et al., 2021). Content analyses revealed substantial overlap between the PROMIS and the EPDS and CES-D, and scores were highly correlated indicating that linkage was appropriate (*r* = 0.81 and 0.94 for EPDS and CES-D, respectively) (Blackwell et al., 2021). Higher PSS t-scores and higher depression t-scores indicate higher perceived stress and depression levels, respectively.

### 2.4. Fetal growth

Fetal growth was assessed using sex-specific birthweight for gestational age z-scores, which were calculated using a US population reference (Talge et al., 2014). Birthweight z-scores are preferred over raw birthweight, as birthweight can be confounded by gestational age at delivery. Information on birthweight and gestational age were abstracted from the medical record in CIOB. In IKIDS, infant birthweight was abstracted from crib cards at the hospital and gestational age was calculated using expected due date reported by the prenatal provider between 16 and 18 weeks (Eick et al., 2021). Due to the small number of small for gestational age births in our study population (N = 56, 6.4%), we focused our analysis on continuous birthweight z-scores.

### 2.5. Covariates

In both cohorts, demographic information was obtained via an interview questionnaire and included maternal age, education, marital status, and race/ethnicity. Information regarding pre-pregnancy body mass index (BMI; kg/m^2^) and parity were abstracted from the medical record in CIOB and were obtained via self-report in IKIDS.

### 2.6. Statistical analysis

Frequencies, counts, means, and standard deviations (SDs) were used to describe our study population. We examined the distribution of individual PFAS using geometric means, geometric SDs and select percentiles. Spearman correlation coefficients (ρ) were used to estimate correlations between PFAS, PSS and depression t-scores, and birthweight z-scores.

We used unadjusted and adjusted linear regression models to examine associations between individual PFAS, measures of psychosocial stress response, and birthweight z-scores. In these models, PFAS and psychosocial stress response measures were standardized to the population’s interquartile range (IQR)/2 to facilitate comparisons with mixture models. To assess potential non-linearity in linear regression models, we fit additional models with categorized values of PFAS and responses to psychosocial stress (with category cut-points at tertiles). Covariates used in adjusted models were chosen via a Directed Acyclic Graph (DAG; Figure S1) (Shrier and Platt, 2008; Hernán et al., 2002), informed by existing literature and correlations between potential confounders and both exposure and outcome variables. Final models included maternal age, race/ethnicity, pre-pregnancy BMI, education, parity, and cohort and were represented using categories outlined in Table 1. Information on experiences of self-reported racism was not available in our analytic sample so we conceptualized race/ethnicity to be a social construct and included it as a proxy for race-based discrimination (Lee et al., 2019). Education was included as an indicator of SES.

### 2.7. Mixtures analysis

We examined the joint effects of PFAS and responses to psychosocial stress in relation to birthweight z-scores using two approaches for exposure mixtures. We first utilized quantile g-computation, which estimates the effect of simultaneously increasing all exposures in the mixture by one quartile using a parametric, generalized linear model-based implementation of g-computation (Keil et al., 2020). In quantile g-computation, PFAS and responses to psychosocial stress are recoded as score variables (0,1,2,…) based on quantile cut-points which are then included in a model for the outcome. This scheme allows each exposure to be given a positive or negative weight, based on the direction of independent association, which are interpreted as the proportion of the partial effect in the positive or negative direction due to a single exposure. Positive and negative weights both sum to 1.0. We considered three mixture groups in our quantile g-computation analysis: 1) PFAS and responses to stress, 2) PFAS alone, and 3) response to stress alone. The mixture model containing all PFAS and responses to stress was considered the “overall” model. In the PFAS alone and response to stress alone models, the remaining PFAS or responses to stress were retained as covariates, respectively.

To further evaluate interactions and potential non-linearity, our second approach utilized Bayesian kernel machine regression (BKMR) (Bobb et al., 2015; Bobb et al., 2018). BKMR estimates a nonparametric high-dimensional exposure–response function using kernel machine regression. We implemented BKMR with component-wide variable selection (20,000 iterations) to account for complex mixtures and to identify potential joint interactions. We checked standard Markov chain Monte Carlo (MCMC) diagnostics to assess convergence (Gelman et al., 2013). In BKMR, importance for each PFAS and stress response measure included in the model was based on posterior inclusion probabilities (PIPs), which correspond to the posterior probability that an exposure is included in the model. For consistency with prior work, a traditional threshold of 0.5 was used (Xu et al., 2021; Cathey et al., 2021; Ashrap et al., 2020; Coker et al., 2018). To examine linearity of individual PFAS and psychosocial stress responses, we examined individual (i.e., univariate) exposure–response functions while holding all other remaining exposures constant at the 50th percentile. Bivariate exposure–response functions were examined to assess interactions between exposures. Evidence of interaction is present if the effect of one exposure differs across levels of another (i.e., the lines are not overlapping). The overall or cumulative effect of the PFAS and response to stress mixture was evaluated by comparing the expected difference in birthweight z-scores when exposures in the mixture were set at the 25th and 75th percentile, as compared to when they were all fixed at their 50th percentile.

We previously observed in the CIOB study population that elevated levels of perceived stress may lead to increased feelings of depression (Eick et al., 2020). Therefore, we conducted sensitivity analysis removing depression t-scores from our overall quantile g-computation model and BKMR analysis. We additionally conducted our mixtures analyses stratified by individual cohort to determine if associations differed across study populations. Mixtures analyses were run on complete cases (N = 876 out of 1,049 total) and all analyses were conducted using R Version 4.1.0 with the packages qgcomp (version 2.8.0) and bkmr (version 0.2.0), respectively.

## 3. Results

The majority of participants included in our analytic sample had a college (28.2%) or graduate degree (40.4%) and were married (74.5%) (Table 1). The mean pre-pregnancy BMI was 26 kg/m^2^ (standard deviation [SD] = 6.1) and the mean maternal age was 32 (SD = 5.1). A higher proportion of IKIDS participants self-identified as white (79.9% in IKIDS vs. 38.7% in CIOB), while more participants in CIOB self-identified as Latina (34.4% in CIOB vs 2.8% in IKIDS). The mean PSS and depression t-scores were slightly higher in CIOB relative to IKIDS (Table 1).

Among the PFAS, the highest geometric mean observed was for PFOS (1.87 ng/mL), followed by PFOA (0.70 ng/mL). For PFAS that were detected in > 70% of participants, the distribution was relatively similar across cohorts. The only exception was for PFUdA, which was detected in 73.9% of CIOB participants compared to only 66% of IKIDS participants (Table 2). PFAS were moderately to strongly correlated with one another and the strongest correlation was between PFDeA and PFUdA (ρ = 0.76) (Figure S2). PSS t-scores and all PFAS were negatively correlated with birthweight z-scores.

In adjusted linear regression models, a one half IQR increase in PFNA (β = −0.01, 95% confidence interval [CI] = −0.05, 0.03), PFOA (β = −0.02, 95% CI = −0.06, 0.03), PFDeA (β = −0.02, 95% CI = −0.06, 0.03), and PFUdA (β = −0.01, 95% CI = −0.06, 0.03) was associated with a slight, non-significant, reduction in birthweight z-scores (Table S2). Increasing depression t-scores were associated with lower birthweight z-scores in linear regression models (β = −0.03, 95% CI = −0.07, 0.00) (Table S3). When modeling tertiles of exposure, the highest compared to lowest tertile of PFNA, PFOA, PFDeA, PFUdA, PSS and depression t-scores was associated with a reduction in birthweight z-scores in unadjusted models only (Table S4).

Using quantile g-computation, increasing all PFAS and responses to stress in the mixture by one quartile was associated with a modest reduction in birthweight z-scores (mean change per quartile increase = −0.09, 95% confidence interval = −0.21, 0.03) (Table 3). This corresponds to a reduction of 39 g for a 40-week gestation birth. In mixture models examining PFAS alone and responses to stress alone, increasing all exposures by one quartile was similarly associated with slight reductions in birthweight z-scores (Table 3). In the overall model, PFDeA and depression t-scores were assigned the largest negative weights (Figure S3). This was similar to what was observed in the PFAS alone and response to stress alone models. The overall mixture effect was slightly attenuated when depression t-scores were removed (mean change in birthweight z-scores per quartile increase = −0.06, 95% CI = −0.18, 0.05) (Table S5). When stratifying by cohort, the overall mixture effect was stronger in IKIDS (mean change in birthweight z-scores per quartile increase = −0.11, 95% CI = −0.26, 0.05) compared to CIOB (mean change in birthweight z-scores per quartile increase = 0.01, 95% CI = −0.18, 0.2) (Table S6). PFDeA was assigned the largest negative weight in quantile g-computation models restricted to CIOB, while PFNA was assigned the largest negative weight in IKIDS (Figure S4).

**Fig. 1.**
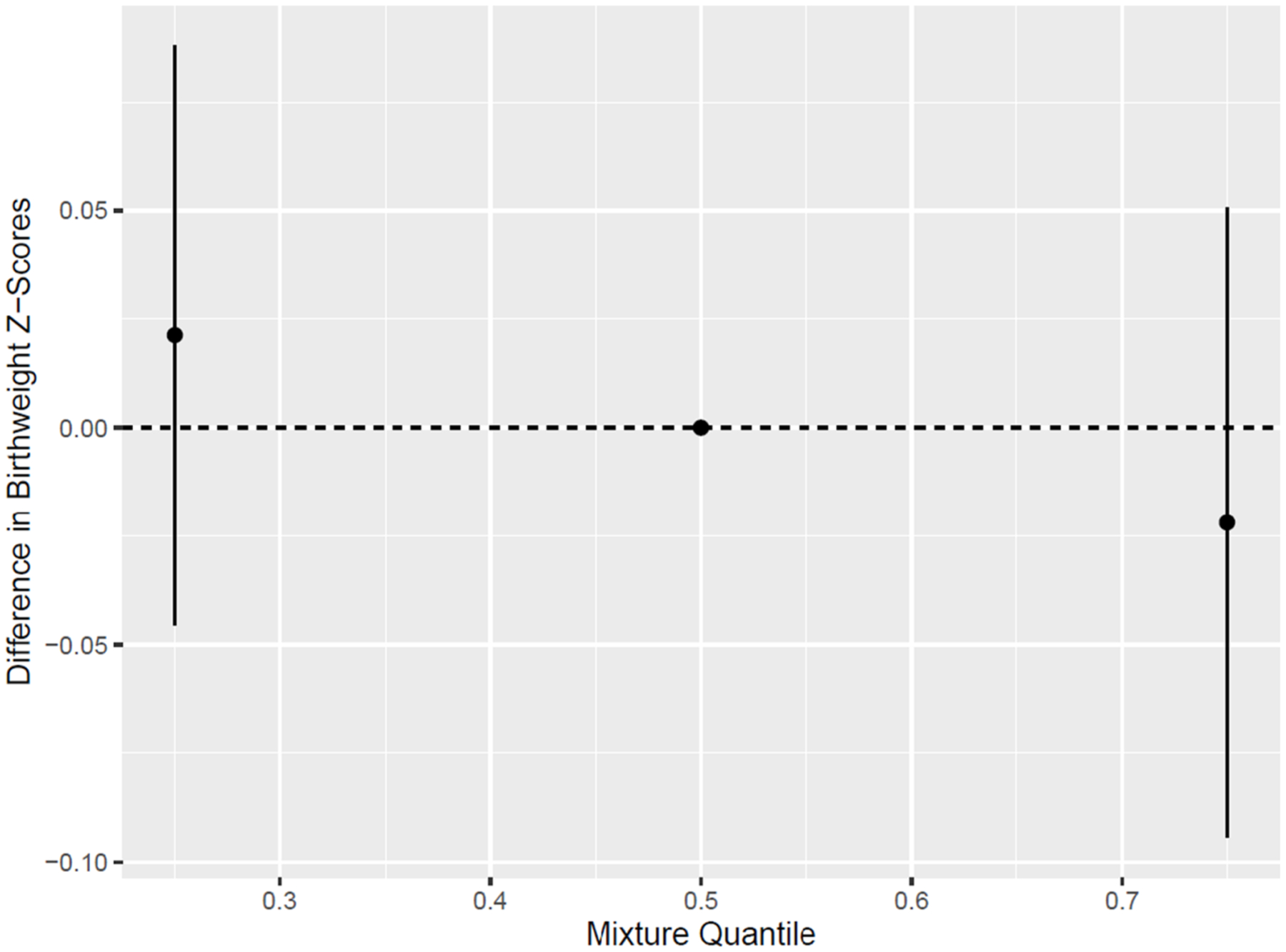
Cumulative effect (estimates and 95% credible intervals) of the PFAS and psychosocial stress response mixture on birthweight z-scores, estimated using BKMR (N = 876). Note: PFAS were natural log transformed. All PFAS and responses to psychosocial stress were scaled to have a mean of 0 and standard deviation of 1. Models are adjusted for maternal education, age, race/ethnicity, pre-pregnancy BMI, parity, and cohort.

**Fig. 2.**
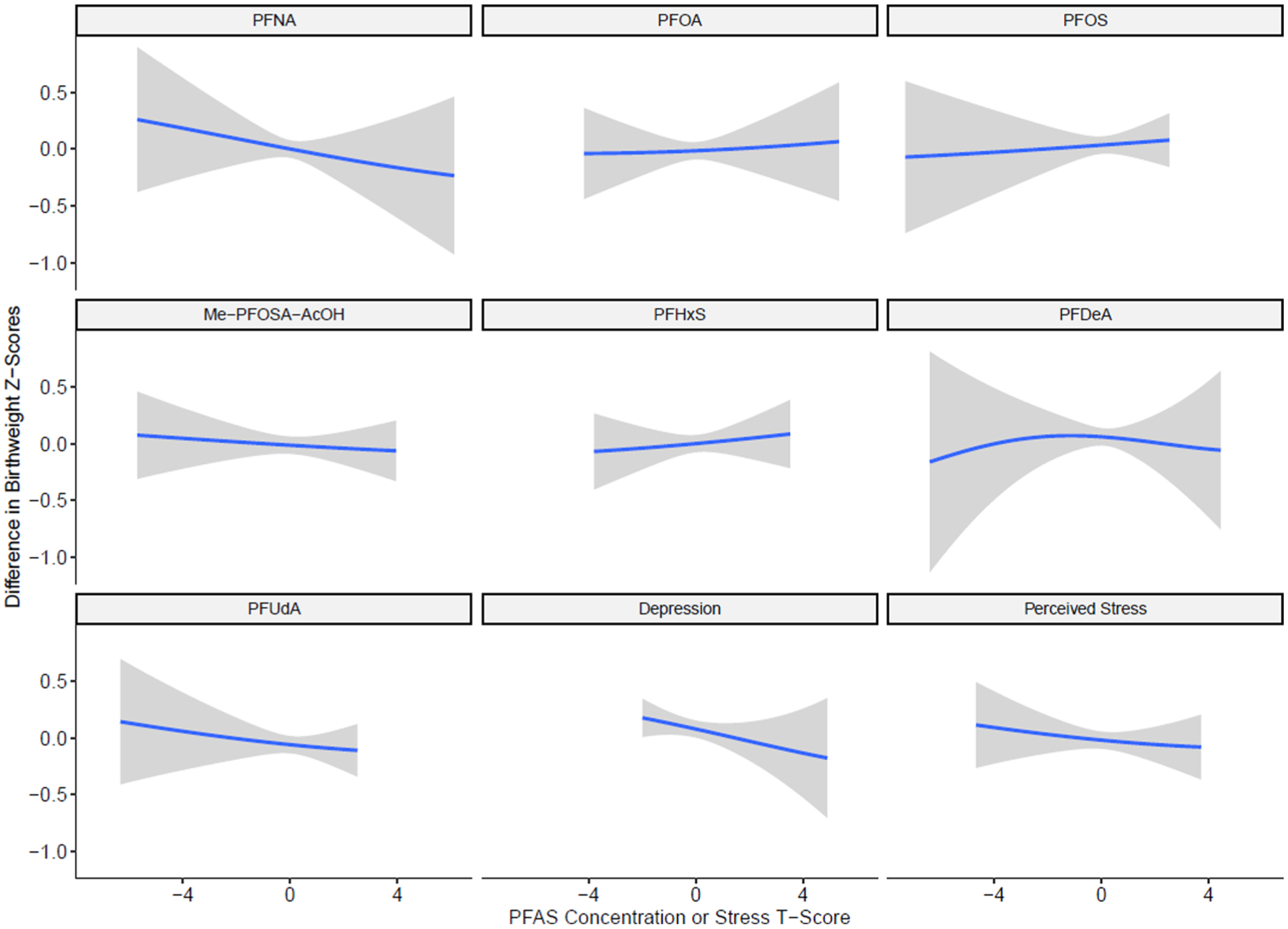
Univariate exposure–response functions and 95% confidence intervals for the change in birthweight z-scores resulting from individual PFAS and response to psychosocial stress while fixing remaining exposures in the mixture at their 50th percentiles, estimated using BKMR (N = 876). Note: PFAS were natural log transformed. All PFAS and responses to psychosocial stress were scaled to have a mean of 0 and standard deviation of 1. Models are adjusted for maternal education, age, race/ethnicity, pre-pregnancy BMI, parity, and cohort.

BKMR analyses did not identify any individual PFAS or response to stress as important individual exposures, as all PIPs were less than 0.5 (Table S7). We did, however, observe that increasing the PFAS and stress mixture was negatively associated with birthweight z-scores (estimate comparing 75th percentile to 50th percentile = −0.02, 95% CI = −0.09, 0.05) (Fig. 1). Our univariate exposure–response analysis showed that PFNA, PFUdA, depression t-scores and PSS t-scores were associated with a slight reduction in birthweight z-scores (Fig. 2). We observed that depression t-scores and PFDeA drove the greatest shifts in effect estimates for all or most other exposures, indicating evidence of potential interaction (Figure S5). When depression t-scores were removed from the BKMR model, the cumulative effect of the overall mixture was stronger (Figure S6). In BKMR analyses stratified by cohort, the cumulative effect of the mixture was negatively associated with birthweight z-scores among IKIDS only (estimate comparing 75th to 50th percentile = −0.06, 95% CI = −0.19, 0.07) (Figure S7). PFHxS, Me-PFOSA-AcOH, perceived stress and depression were identified as important individual exposures in CIOB only (PIP > 0.5) (Table S8).

## 4. Discussion

We investigated the joint effects of prenatal exposure to multiple PFAS, perceived stress, and depression on fetal growth using two mixture methods. In our large, demographically diverse cohort of pregnant people in San Francisco, CA and Urbana-Champaign, IL, we found that increasing PFAS and stress mixtures were associated with modest reductions in birthweight z-scores, although confidence intervals included the null value. Both quantile g-computation and BKMR indicated that the cumulative association was due to multiple exposures and did not identify a single bad actor. The combined change in birthweight z-scores resulting from both PFAS and stress exposures in the overall model was stronger than the individual effect of either chemical or stress response measurement group, which underscores the utility of mixture methods to assess chemical and non-chemical stressors simultaneously.

Our study is one of a few to apply mixture methods to examine the joint effects of both chemical and non-chemical stressors during pregnancy and the methods applied in our study may serve as a template for future work. Among participants in the NICHD Fetal Growth Study, no association was observed between mixtures of heavy metals (lead, mercury, and cadmium measured in maternal plasma), PSS, and depression and small for gestational age (SGA) births using Weighted Quantile Sum (WQS) regression (Zilversmit Pao et al., 2019). This is contrast to our findings that chemicals and stressors have a cumulative effect on fetal growth. Differences may be attributed to differences in mixture methods, as quantile g-computation may be preferred over WQS regression, as it allows for bi-directional exposure-outcome relationships. We also acknowledge that results from our study may not be directly comparable to the NICHD Fetal Growth study, as the chemical exposures differed. An additional study of pregnant people in the Caribbean utilized a path analysis, a subset of structural equation modeling (Gokoel et al., 2021). In that study, the chemical construct (comprised of mercury, lead, selenium, and tin measured in maternal whole blood) and increasing PSS and depression scores were associated with lower birthweight and gestational age (Gokoel et al., 2021), which supports our findings that there is a joint effect of chemical and non-chemical stressors on fetal growth. Other studies using stratified single pollutant models have shown that the relationships between increasing chemical exposures and adverse pregnancy outcomes are stronger in the presence of stress (Padula et al., 2020; Aker et al., 2020; Ferguson et al., 2019; Ashrap et al., 2021).

Both of our mixture approaches indicated that there was a modest reduction in birthweight z-scores in relation to increasing PFAS exposure, although confidence intervals were wide. Other epidemiologic studies have produced more consistent findings and have shown that increasing exposure to PFOA, PFOS, PFNA, and PFDeA is associated with lower infant birthweight (Yao et al., 2021; Zhu and Bartell, 2020; Kashino et al., 2020; Rokoff et al., 2018). This is supported by animal studies, which find that exposure to PFOA, PFOS, and PFNA *in utero* is associated with lower offspring birthweight (Grasty et al., 2003; Luebker et al., 2005; Wolf et al., 2007; Wolf et al., 2010). However, PFAS exposure levels used in these experimental animal studies are substantially higher than PFAS levels observed in our study. An additional study utilizing WQS regression to examine mixture effects of multiple endocrine disrupting chemicals and fetal growth, identified PFOA and PFDA as predictors of lower birthweight z-scores (Svensson et al., 2021). A recent retrospective ecologic study in Italy further found that the prevalence of SGA births was higher in PFAS-contaminated regions relative to non-contaminated areas (Manea et al., 2020). While other studies, including systematic reviews have found null associations between PFAS and fetal growth or birthweight (Steenland et al., 2018; Dzierlenga et al., 2020; Gao et al., 2021; Chen et al., 2012; Meng et al., 2018; Costa et al., 2019), these studies have mostly used single pollutant linear regression models and suggest that associations may vary based on when PFAS were measured during pregnancy. In our adjusted linear regression models for the association between individual PFAS and birthweight z-scores all confidence intervals included the null value. All PFAS included in our analyses were long-chain PFAS and may indicate that these PFAS have similar health effects. This may be one reason why linear regression and mixture methods revealed no single ‘bad actor.’ While point estimates obtained from quantile g-computation also included the null value, the point estimates were greater in magnitude when PFAS were modeled in conjunction with stress as an exposure mixture. This highlights the importance of considering an exposure mixture that incorporates non-chemical stressors, as it allowed us to elucidate relationships that otherwise would have been missed.

In our study population, PFAS levels were lower than what was observed among reproductive age women in NHANES from 2013 to 2014 (Eick et al., 2021). This could be due to levels of some legacy PFAS, including PFOA and PFOS, declining over time because of industry phase outs (Brennan et al., 2021; Shu et al., 2018). Levels in our study may also have declined with increasing parity, as many of our study participants have had at least one prior birth and PFAS are excreted through breastmilk (Mogensen et al., 2015). Nonetheless, PFAS levels in our study population were elevated among higher SES individuals (Eick et al., 2021). This could be attributed to exposure from home improvement and other consumer products or dietary patterns, as we have previously found these to be sources of PFAS exposure in the CIOB cohort (Eick et al., 2021; Wang et al., 2021). In this prior work, we found that PFDeA and PFUdA were the two PFAS most strongly associated with fish, poultry, and red meat consumption (Eick et al., 2021). Participants in CIOB may have had different dietary patterns than IKIDS participants as a result of living in different geographic regions, which could be one reason for the higher PFDeA and PFUdA concentrations observed in CIOB relative to IKIDS.

We observed that increasing PSS and depression t-scores were associated with a slight, non-significant, reduction in birthweight z-scores using quantile g-computation and BKMR. These findings are supported by prior work that has found prenatal stress to be a risk factor for lower birthweight. For example, the upper two quantiles of scores on the PSS, administered during mid-pregnancy, were associated low birthweight relative to scores in the lowest quantile (Szegda et al., 2018). Increasing perceived stress and depression were similarly associated with lower birthweight among Mexican American women (Ruiz et al., 2021). In contrast, the NICHD fetal growth study found no strong associations between perceived stress or depression and fetal growth, as measured using sonographic data with individual biometric parameters (Grobman et al., 2017). These differences could be due to how fetal growth was defined, as we used birthweight z-scores as a measure of fetal growth in our study.

Biologically, the PFAS and stressors examined in our study may influence fetal growth individually and jointly through multiple different pathways (Padula et al., 2020; Barrett and Padula, 2019). Environmental chemicals enter the maternal circulation, leading to endocrine disruption, systemic inflammation, metabolic dysfunction and epigenetic changes via the HPA axis, which is also sensitive to psychosocial stress exposures (Padula et al., 2020; Barrett and Padula, 2019). Upstream social and demographic factors, such as segregation and immigration status, can influence psychosocial stress and chemical exposure levels, indicating common exposure pathways (Padula et al., 2020; Barrett and Padula, 2019). For example, we previously found that increasing exposure to PFNA, PFOA, and perceived stress was associated with elevated CRH in the CIOB cohort (Eick et al., 2022). In this prior work, the relationship between PFNA and CRH was stronger among women who experienced stress, indicating a joint effect of chemical and non-chemical stressors on CRH (Eick et al., 2022). Other pathways may be through inflammation, as studies have linked depression to elevated maternal biomarkers of inflammation and recent work suggests that PFAS are also associated with chronic inflammation (Omoike et al., 2021; Lahti-Pulkkinen et al., 2020).

An important strength of our study is that we examined the joint effects of PFAS and non-chemical stressors using two mixture methods. Our application of mixture methods also allows us to better understand real life exposures, as individuals are exposed to numerous chemical and non-chemical stressors simultaneously. Additionally, our study population was demographically diverse and spanned two geographic regions in the US. Results from our study can provide important insights into populations routinely underrepresented in epidemiologic studies but may not be reflective of highly exposed populations. We also acknowledge the limitations of our study. We did not have information on history of breastfeeding, which may influence PFAS levels, as PFAS are excreted through breastmilk. However, we did retain parity as a covariate in adjusted models, which may serve as a proxy for prior experiences of breastfeeding (Hackman et al., 2015). While we used current best practices to harmonize stressors using t-scores, participants may respond differently to individual questions on the stress questionnaires including 4 vs 10 questions on the PSS and using different depression scales across cohorts to assess depression. Information on maternal history of depression was also unavailable in our study, potentially resulting in differential misclassification of stress exposures. Further, information on perceived discrimination was not available in our study population. We conceptualized race/ethnicity as a social construct reflecting experiences of discrimination and material hardship in adjusted models, however we acknowledge that this proxy may not truly reflect reality as individuals may experience discrimination differently. Additionally, while our study population was demographically diverse, most participants were highly educated, which may limit our generalizability. Our wide confidence intervals may also reflect low statistical power; in analyses restricted to individual cohorts, the mixture was negatively associated with birthweight z-scores only among IKIDS participants, which likely is a result of modestly higher concentrations of commonly detected PFAS (PFOA, PFOS, PFHxS) relative to CIOB. Approximately 40% of the CIOB study population was born outside of the US and foreign-born participants had lower PFAS level than those born in the US, which may be another reason why results obtained from mixture models differed across cohorts. Lastly, as with all observational studies, our results may be subject to residual confounding.

## 5. Conclusions

Our study is one of the first studies applying environmental mixture methods to chemical and non-chemical stress exposures. We found that prenatal exposure to PFAS and perceived stress, and depression, modeled together as a mixture, was modestly associated with lower birthweight z-scores. The effects of PFS and stressors in combination was greater than what was observed with individual exposures. The mixture methods applied in our study suggested that PFAS and responses to stress were both important to fetal growth. Future studies should consider whether non-chemical stressors can increase the susceptibility to environmental chemical exposures.

## Supplementary Material

Supplementary Material

## Figures and Tables

**Table 1:** Distribution of ECHO.CA.IL analytic sample.

	CIOB (N = 622)	IKIDS (N = 427)	Total (N = 1049)
**Maternal Age at Delivery (years)**			
Mean (SD)	33 (5.4)	30 (4.2)	32 (5.1)
**Pre-pregnancy Body Mass Index (kg/m2)**			
Mean (SD)	26 (5.4)	27 (6.8)	26 (6.1)
Missing	59 (9.5%)	4 (0.9%)	63 (6.0%)
**Gestational Age (weeks)**			
Mean (SD)	39 (1.9)	39 (1.2)	39 (1.7)
**Birth Weight (grams)**			
Mean (SD)	3353 (575)	3482 (436)	3408 (524)
	N (%)	N (%)	N (%)
**Maternal Education**			
Less than College	225 (36.2%)	83 (19.4%)	308 (29.4%)
College Degree	139 (22.4%)	156 (36.5%)	296 (28.2%)
Graduate Degree	237 (38.1%)	188 (44.0%)	424 (40.4%)
Missing	21 (3.4%)	0 (0%)	21 (2.0%)
**Maternal Race/Ethnicity**			
White	241 (38.7%)	341 (79.9%)	582 (55.5%)
Black	37 (6%)	24 (5.6%)	61 (5.8%)
Asian/Pacific Islander	109 (17.5%)	22 (5.2%)	131 (12.5%)
Latina	214 (34.4%)	12 (2.8%)	226 (21.5%)
Other/Multi-Racial	16 (2.6%)	28 (6.6%)	44 (4.2%)
Missing	5 (0.8%)	0 (0%)	5 (0.5%)
**Infant Sex**			
Male	301 (48.4%)	215 (50.4%)	516 (49.2%)
Female	321 (51.6%)	212 (49.6%)	533 (50.8%)
**Parity**			
1 + Births	316 (50.8%)	260 (60.9%)	576 (54.9%)
No Prior Births	299 (48.1%)	167 (39.1%)	466 (44.4%)
Missing	7 (1.1%)	0 (0%)	7 (0.7%)
**Marital Status**			
Married	410 (65.9%)	372 (87.1%)	782 (74.5%)
Living Together	119 (19.1%)	32 (7.5%)	151 (14.4%)
Single	62 (10.0%)	23 (5.4%)	84 (8.0%)
Missing	31 (5.0%)	0 (0%)	32 (3.1%)
**Perceived Stress T-Score**			
Mean (SD)	50 (8.5)	45 (11)	48 (10)
Missing	36 (5.8%)	6 (1.4%)	42 (4.0%)
**Depression T-Score**			
Mean (SD)	48 (5.5)	46 (8.0)	47 (6.7)
Missing	67 (10.8%)	3 (0.7%)	70 (6.7%)

Abbreviations: SD, standard deviation.

**Table 2:** Distribution of second trimester serum levels of per- and polyfluoroalkyl substances (ng/mL) in the ECHO.CA.IL analytic sample (N = 1,049)

	% Above MDL	% Machine Readable	Geometric Mean (Geometric SD)	Percentile				
				5th	25th	50th	75th	95th
PFNA								
Overall	98.57	99.71	0.28 (1.98)	0.09	0.18	0.29	0.42	0.78
CIOB	98.71	99.52	0.28 (1.97)	0.09	0.19	0.29	0.43	0.81
IKIDS	98.36	100	0.27 (1.99)	0.09	0.18	0.29	0.42	0.75
PFOA								
Overall	99.52	99.9	0.7 (2.06)	0.22	0.44	0.73	1.16	2.11
CIOB	99.36	99.84	0.69 (2.05)	0.23	0.44	0.72	1.1	2.04
IKIDS	99.77	100	0.72 (2.08)	0.21	0.43	0.73	1.2	2.46
PFHxS								
Overall	99.24	99.52	0.39 (2.59)	0.09	0.22	0.38	0.71	1.89
CIOB	99.52	99.84	0.33 (2.45)	0.09	0.19	0.31	0.56	1.47
IKIDS	98.83	99.06	0.5 (2.66)	0.11	0.29	0.51	0.94	2.33
PFOS								
Overall	99.52	99.81	1.87 (2.2)	0.54	1.2	1.98	3.12	6.13
CIOB	99.84	99.84	1.74 (2.09)	0.5	1.1	1.78	2.92	5.66
IKIDS	99.06	99.77	2.08 (2.32)	0.62	1.4	2.2	3.52	6.85
Me-PFOSA-AcOH								
Overall	85.32	95.42	0.04 (3.22)	0.01	0.02	0.04	0.08	0.22
CIOB	85.85	95.82	0.04 (2.77)	0.01	0.02	0.04	0.07	0.15
IKIDS	84.54	94.85	0.04 (3.89)	0.01	0.02	0.05	0.1	0.35
PFDeA								
Overall	69.88	95.33	0.1 (2.32)	0.02	0.06	0.1	0.17	0.32
CIOB	70.42	93.73	0.11 (2.27)	0.03	0.07	0.12	0.19	0.36
IKIDS	69.09	97.66	0.08 (2.28)	0.02	0.05	0.08	0.14	0.26
PFUdA								
Overall	70.64	93.99	0.06 (3.29)	0.01	0.03	0.07	0.14	0.33
CIOB	73.79	95.5	0.09 (2.83)	0.02	0.04	0.1	0.18	0.38
IKIDS	66.04	91.8	0.04 (3.42)	0.01	0.02	0.05	0.09	0.2
PFDoA								
Overall	2.38	59.39	0.04 (4.12)	0.003	0.02	0.06	0.1	0.14
CIOB	2.25	61.41	0.05 (3.76)	0.004	0.02	0.05	0.14	0.14
IKIDS	2.58	56.44	0.03 (4.38)	0.002	0.01	0.06	0.08	0.08
PFOSA								
Overall	6.86	51.29	0.01 (2.99)	0.001	0.01	0.01	0.02	0.02
CIOB	3.05	49.04	0.01 (3.4)	0.001	0.004	0.01	0.02	0.02
IKIDS	12.41	54.57	0.01 (2.38)	0.001	0.01	0.01	0.01	0.03
PFBS								
Overall	1.53	44.52	0.01 (2.72)	0.001	0.01	0.02	0.02	0.02
CIOB	2.09	47.59	0.01 (2.95)	0.001	0.01	0.02	0.02	0.02
IKIDS	0.7	40.05	0.01 (2.36)	0.002	0.01	0.02	0.02	0.02
Et-PFOSA-AcOH								
Overall	9.53	54.53	0.01 (2.54)	0.001	0.01	0.01	0.01	0.02
CIOB	8.36	63.67	0.01 (2.81)	0.001	0.004	0.01	0.01	0.02
IKIDS	11.24	41.22	0.01 (2.04)	0.003	0.01	0.01	0.01	0.02
PFHpA								
Overall	15.16	65.49	0.02 (2.87)	0.003	0.01	0.02	0.04	0.07
CIOB	14.31	70.74	0.02 (3.01)	0.003	0.01	0.03	0.04	0.06
IKIDS	16.39	57.85	0.02 (2.64)	0.003	0.01	0.02	0.02	0.07

Note: Geometric mean, geometric SD, and percentile values use the machine read value if it was available. If there was no machine read value, missing values were replaced with MDL/square root of 2.

Abbreviations: SD, standard deviation, MDL, method detection limit.

**Table 3:** Quantile g-computation estimates and 95% confidence intervals for the change in birthweight z-scores for a one quartile increase in the mixture of PFAS and responses to psychosocial stress (N = 876).

	β	(95% CI)
^1^Overall	−0.09	(−0.21, 0.03)
^2^PFAS	−0.03	(−0.13, 0.07)
^3^Psychosocial Stress Response	−0.06	(−0.13, 0.01)

Note: Beta estimates are interpreted as the effect on birthweight z-scores of increasing every exposure in the mixture by one quantile.

1 Models are adjusted for maternal education, age, race/ethnicity, pre-pregnancy BMI, parity, and cohort.

2 Mixture effect is for only PFAS with ≥ 70% detection (PFNA, PFOS, PFOA, PFHxS, PFDeA, PFUdA, and Me-PFOSA-AcOH), adjusted for other stressors, maternal education, age, race/ethnicity, pre-pregnancy BMI, parity, and cohort.

3 Mixture effect is for only responses to psychosocial stressors (perceived stress, depression), adjusted for other PFAS with ≥ 70% detection, maternal education, age, race/ethnicity, pre-pregnancy BMI, parity, and cohort.

Abbreviations: CI, confidence interval.

## References

[R1] Aker A, McConnell RER, Loch-Caruso R, Park SK, Mukherjee B, Rosario ZY, Vélez-Vega CM, Huerta-Montanez G, Alshawabkeh AN, Cordero JF, Meeker JD, 2020. Interactions between chemicals and non-chemical stressors: The modifying effect of life events on the association between triclocarban, phenols and parabens with gestational length in a Puerto Rican cohort. Sci Total Environ. 708 10.1016/j.scitotenv.2019.134719. PMC6957748 31785910

[R2] Andrews DQ, Naidenko OV, 2020. Population-Wide Exposure to Per- and Polyfluoroalkyl Substances from Drinking Water in the United States. Environ Sci Technol Lett. 7 (12), 931–936. 10.1021/acs.estlett.0c00713.

[R3] Ashrap P, Watkins DJ, Mukherjee B, Boss J, Richards MJ, Rosario Z, Vélez-Vega CM, Alshawabkeh A, Cordero JF, Meeker JD, 2020. Maternal blood metal and metalloid concentrations in association with birth outcomes in Northern Puerto Rico. Environ Int. 138, 105606. 32179314 10.1016/j.envint.2020.105606 PMC7198231

[R4] Ashrap P, Aker A, Watkins DJ, Mukherjee B, Rosario-Pabón Z, Vélez-Vega CM, Alshawabkeh A, Cordero JF, Meeker JD, 2021. Psychosocial status modifies the effect of maternal blood metal and metalloid concentrations on birth outcomes. Environ. Int 149 10.1016/j.envint.2021.106418. PMC7897320 33548848

[R5] Barrett ES, Padula AM, 2019. Joint Impact of Synthetic Chemical and Non-chemical Stressors on Children’s Health. Curr Environ Health Rep. 6 (4), 225–235. 10.1007/s40572-019-00252-6. 31637664 PMC6923601

[R6] Blackwell CK, Tang X, Elliott AJ, Thomes T, Louwagie H, Gershon R, Schalet BD, Cella D, 2021. Developing a common metric for depression across adulthood: Linking PROMIS depression with the Edinburgh Postnatal Depression Scale. Psychol. Assess 33 (7), 610–618. 34060864 10.1037/pas0001009 PMC8284177

[R7] Bobb JF, Valeri L, Claus Henn B, Christiani DC, Wright RO, Mazumdar M, Godleski JJ, Coull BA, 2015. Bayesian kernel machine regression for estimating the health effects of multi-pollutant mixtures. Biostatistics. 16 (3), 493–508. 25532525 10.1093/biostatistics/kxu058 PMC5963470

[R8] Bobb JF, Claus Henn B, Valeri L, Coull BA, 2018. Statistical software for analyzing the health effects of multiple concurrent exposures via Bayesian kernel machine regression. Environmental Health. 17 (1), 67. 10.1186/s12940-018-0413-y. 30126431 PMC6102907

[R9] Brennan NM, Evans AT, Fritz MK, Peak SA, von Holst HE, 2021. Trends in the Regulation of Per- and Polyfluoroalkyl Substances (PFAS): A Scoping Review. Int J Environ Res Public Health. 18 (20), 10900. 10.3390/ijerph182010900. 34682663 PMC8536021

[R10] Calafat AM, Wong L-Y, Kuklenyik Z, Reidy JA, Needham LL, 2007. Polyfluoroalkyl Chemicals in the U.S. Population: Data from the National Health and Nutrition Examination Survey (NHANES) 2003–2004 and Comparisons with NHANES 1999–2000. Environ. Health Perspect 115 (11), 1596–1602. 18007991 10.1289/ehp.10598 PMC2072821

[R11] Cathey AL, Eaton JL, Ashrap P, Watkins DJ, Rosario ZY, Vélez Vega C, Alshawabkeh AN, Cordero JF, Mukherjee B, Meeker JD, 2021. Individual and joint effects of phthalate metabolites on biomarkers of oxidative stress among pregnant women in Puerto Rico. Environ. Int 154 10.1016/j.envint.2021.106565. PMC9923976 33882432

[R12] Chen M-H, Ha E-H, Wen T-W, Su Y-N, Lien G-W, Chen C-Y, Chen P-C, Hsieh W-S, Meliker J, 2012. Perfluorinated compounds in umbilical cord blood and adverse birth outcomes. PLoS ONE 7 (8). 10.1371/journal.pone.0042474. PMC3411780 22879996

[R13] Clarity C, Trowbridge J, Gerona R, Ona K, McMaster M, Bessonneau V, Rudel R, Buren H, Morello-Frosch R, 2021. Associations between polyfluoroalkyl substance and organophosphate flame retardant exposures and telomere length in a cohort of women firefighters and office workers in San Francisco. Environ Health. 20 (1) 10.1186/s12940-021-00778-z. PMC8403436 34454526

[R14] Clougherty JE, Shmool JLC, Kubzansky LD, 2014. The Role of Non-Chemical Stressors in Mediating Socioeconomic Susceptibility to Environmental Chemicals. Current Environmental Health Reports. 1 (4), 302–313. 10.1007/s40572-014-0031-y.

[R15] Cohen S, Kamarck T, Mermelstein R, 1983. A global measure of perceived stress. J Health Soc Behav. 24 (4), 385–396. 6668417

[R16] Cohen S Perceived stress in a probability sample of the United States. In: The Social Psychology of Health. The Claremont Symposium on Applied Social Psychology. Sage Publications, Inc; 1988:31–67.

[R17] Coker E, Chevrier J, Rauch S, Bradman A, Obida M, Crause M, Bornman R, Eskenazi B, 2018. Association between prenatal exposure to multiple insecticides and child body weight and body composition in the VHEMBE South African birth cohort. Environ Int. 113, 122–132. 29421401 10.1016/j.envint.2018.01.016 PMC5866210

[R18] Costa O, Iñiguez C, Manzano-Salgado CB, Amiano P, Murcia M, Casas M, Irizar A, Basterrechea M, Beneito A, Schettgen T, Sunyer J, Vrijheid M, Ballester F, Lopez-Espinosa M-J, 2019. First-trimester maternal concentrations of polyfluoroalkyl substances and fetal growth throughout pregnancy. Environ. Int 130 10.1016/j.envint.2019.05.024. 31247476

[R19] Cox JL, Holden JM, Sagovsky R, 1987. Detection of postnatal depression. Development of the 10-item Edinburgh Postnatal Depression Scale. Br J Psychiatry. 150, 782–786. 10.1192/bjp.150.6.782. 3651732

[R20] Deji Z, Liu P, Wang X, Zhang X, Luo Y, Huang Z, 2021. Association between maternal exposure to perfluoroalkyl and polyfluoroalkyl substances and risks of adverse pregnancy outcomes: A systematic review and meta-analysis. Sci. Total Environ 783, 146984 10.1016/j.scitotenv.2021.146984. 34088118

[R21] Dole N, Savitz DA, Hertz-Picciotto I, Siega-Riz AM, McMahon MJ, Buekens P, 2003. Maternal Stress and Preterm Birth. Am. J. Epidemiol 157 (1), 14–24. 10.1093/aje/kwf176. 12505886

[R22] Dzierlenga MW, Crawford L, Longnecker MP. Birth weight and perfluorooctane sulfonic acid: a random-effects meta-regression analysis. Environmental Epidemiology. 2020;4 (3). https://journals.lww.com/environepidem/Fulltext/2020/06000/Birth_weight_and_perfluorooctane_sulfonic_acid__a.2.aspx. 10.1097/EE9.0000000000000095 PMC7941775 33778349

[R23] Eick SM, Goin DE, Trowbridge J, Dietary predictors of prenatal per- and polyfluoroalkyl substances exposure. Journal of Exposure Science & Environmental Epidemiology. Published online October 6, 2021. doi:10.1038/s41370-021-00386-6. PMC8983786 34615969

[R24] Eick SM, Hom Thepaksorn EK, Izano MA, Cushing LJ, Wang Y, Smith SC, Gao S, Park J-S, Padula AM, DeMicco E, Valeri L, Woodruff TJ, Morello-Frosch R, 2020. Associations between prenatal maternal exposure to per- and polyfluoroalkyl substances (PFAS) and polybrominated diphenyl ethers (PBDEs) and birth outcomes among pregnant women in San Francisco. Environ Health. 19 (1) 10.1186/s12940-020-00654-2. PMC7495899 32938446

[R25] Eick SM, Goin DE, Izano MA, Cushing L, DeMicco E, Padula AM, Woodruff TJ, Morello-Frosch R, Hashimoto K, 2020. Relationships between psychosocial stressors among pregnant women in San Francisco: A path analysis. PLoS ONE 15 (6). 10.1371/journal.pone.0234579. PMC7292353 32530956

[R26] Eick SM, Enright EA, Geiger SD, Dzwilewski KLC, DeMicco E, Smith S, Park J-S, Aguiar A, Woodruff TJ, Morello-Frosch R, Schantz SL, 2021;18(2): 742.. Associations of Maternal Stress, Prenatal Exposure to Per- and Polyfluoroalkyl Substances (PFAS), and Demographic Risk Factors with Birth Outcomes and Offspring Neurodevelopment: An Overview of the ECHO.CA.IL Prospective Birth Cohorts. Int. J. Environ. Res. Public Health 18 (2), 742. 10.3390/ijerph18020742 PMC7830765 33467168

[R27] Eick SM, Goin DE, Cushing L, DeMicco E, Smith S, Park J-S, Padula AM, Woodruff TJ, Morello-Frosch R, 2022. Joint effects of prenatal exposure to per- and poly-fluoroalkyl substances and psychosocial stressors on corticotropin-releasing hormone during pregnancy. J Expo Sci Environ Epidemiol 32 (1), 27–36. 33824413 10.1038/s41370-021-00322-8 PMC8492777

[R28] Ferguson KK, Rosen EM, Barrett ES, Nguyen RHN, Bush N, McElrath TF, Swan SH, Sathyanarayana S, 2019. Joint impact of phthalate exposure and stressful life events in pregnancy on preterm birth. Environ. Int 133 10.1016/j.envint.2019.105254. PMC6924167 31675562

[R29] Gao X, Ni W, Zhu S, Wu Y, Cui Y, Ma J, Liu Y, Qiao J, Ye Y, Yang P, Liu C, Zeng F, 2021. Per- and polyfluoroalkyl substances exposure during pregnancy and adverse pregnancy and birth outcomes: A systematic review and meta-analysis. Environ. Res 201 10.1016/j.envres.2021.111632. 34237336

[R30] Gelman A, Carlin JB, Stern HS, Vehtari A, Rubin DB. Bayesian Data Analysis. 3rd ed. Chapman and Hall/CRC; 2013.

[R31] Goin DE, Izano MA, Eick SM, Maternal experience of multiple hardships and fetal growth: Extending environmental mixtures methodology to social exposures. Epidemiology. Published online October 5, 2020. doi:10.1097/ede.0000000000001272. PMC7708528 33031217

[R32] Gokoel AR, Shankar A, Abdoel Wahid F, Hindori-Mohangoo AD, Covert HH, Wickliffe JK, Harville EW, Zijlmans WCWR, Lichtveld MY, 2021. The Cumulative Risk of Prenatal Exposures to Chemical and Non-Chemical Stressors on Birth Outcomes in Suriname. Int J Environ Res Public Health. 18 (14), 7683. 34300134 10.3390/ijerph18147683 PMC8305475

[R33] Grasty RC, Grey BE, Lau CS, Rogers JM, 2003. Prenatal window of susceptibility to perfluorooctane sulfonate-induced neonatal mortality in the Sprague-Dawley rat. Birth Defects Res. B 68 (6), 465–471. 10.1002/bdrb.10046. 14745980

[R34] Grobman WA, Wing DA, Albert P, Kim S, Grewal J, Guille C, Newman R, Chien EK, Owen J, D’Alton ME, Wapner R, Sciscione A, Grantz KL, 2017. Maternal Depressive Symptoms, Perceived Stress, and Fetal Growth. J Ultrasound Med. 36 (8), 1639–1648. 28393386 10.7863/ultra.16.08085 PMC5967616

[R35] Hackman NM, Schaefer EW, Beiler JS, Rose CM, Paul IM, 2015. Breastfeeding outcome comparison by parity. Breastfeed Med. 10 (3), 156–162. 10.1089/bfm.2014.0119. 25549051 PMC4378341

[R36] Hernán MA, Hernández-Díaz S, Werler MM, Mitchell AA, 2002. Causal Knowledge as a Prerequisite for Confounding Evaluation: An Application to Birth Defects Epidemiology. Am. J. Epidemiol 155 (2), 176–184. 10.1093/aje/155.2.176. 11790682

[R37] Hornung RW, Reed LD, 1990. Estimation of Average Concentration in the Presence of Nondetectable Values. null. 5 (1), 46–51. 10.1080/1047322X.1990.10389587.

[R38] Kang H, Kim H-S, Yoon YS, Lee J, Kho Y, Lee J, Chang HJ, Cho YH, Kim YA, 2021. Placental Transfer and Composition of Perfluoroalkyl Substances (PFASs): A Korean Birth Panel of Parent-Infant Triads. Toxics. 9 (7), 168. 34357911 10.3390/toxics9070168 PMC8309930

[R39] Kashino I, Sasaki S, Okada E, Matsuura H, Goudarzi H, Miyashita C, Okada E, Ito YM, Araki A, Kishi R, 2020. Prenatal exposure to 11 perfluoroalkyl substances and fetal growth: A large-scale, prospective birth cohort study. Environ. Int 136 10.1016/j.envint.2019.105355. 32029284

[R40] Keil AP, Buckley JP, O’Brien KM, Ferguson KK, Zhao S, White AJ, 2020. A Quantile-Based g-Computation Approach to Addressing the Effects of Exposure Mixtures. Environ Health Perspect. 128 (4), 47004. 10.1289/ehp5838. 32255670 PMC7228100

[R41] Kupst MJ, Butt Z, Stoney CM, Griffith JW, Salsman JM, Folkman S, Cella D, 2015. Assessment of stress and self-efficacy for the NIH Toolbox for Neurological and Behavioral Function. Anxiety Stress Coping. 28 (5), 531–544. 25577948 10.1080/10615806.2014.994204 PMC4515370

[R42] Lahti-Pulkkinen M, Girchenko P, Robinson R, Lehto SM, Toffol E, Heinonen K, Reynolds RM, Kajantie E, Laivuori H, Villa PM, Hämäläinen E, Lahti J, Räikkönen K, 2020. Maternal depression and inflammation during pregnancy. Psychol. Med 50 (11), 1839–1851. 31439060 10.1017/S0033291719001909

[R43] Latendresse G, 2009. The interaction between chronic stress and pregnancy: preterm birth from a biobehavioral perspective. J Midwifery Womens Health. 54 (1), 8–17. 10.1016/j.jmwh.2008.08.001. 19114234 PMC2651684

[R44] Lee RT, Perez AD, Boykin CM, Mendoza-Denton R, 2019. On the prevalence of racial discrimination in the United States. PLoS ONE 14 (1). 10.1371/journal.pone.0210698. PMC6328188 30629706

[R45] Luebker DJ, York RG, Hansen KJ, Moore JA, Butenhoff JL, 2005. Neonatal mortality from in utero exposure to perfluorooctanesulfonate (PFOS) in Sprague-Dawley rats: Dose–response, and biochemical and pharamacokinetic parameters. Toxicology 215 (1), 149–169. 10.1016/j.tox.2005.07.019. 16129535

[R46] Manea S, Salmaso L, Lorenzoni G, Mazzucato M, Russo F, Mantoan D, Martuzzi M, Fletcher T, Facchin P, 2020. Exposure to PFAS and small for gestational age new-borns: A birth records study in Veneto Region (Italy). Environ. Res 184 10.1016/j.envres.2020.109282. 32120121

[R47] Meng Q, Inoue K, Ritz B, Olsen J, Liew Z, 2018. Prenatal Exposure to Perfluoroalkyl Substances and Birth Outcomes; An Updated Analysis from the Danish National Birth Cohort. Int J Environ Res Public Health. 15 (9) 10.3390/ijerph15091832. PMC6164159 30149566

[R48] Mogensen UB, Grandjean P, Nielsen F, Weihe P, Budtz-Jørgensen E, 2015. Breastfeeding as an Exposure Pathway for Perfluorinated Alkylates. Environ Sci Technol. 49 (17), 10466–10473. 10.1021/acs.est.5b02237. 26291735 PMC6190571

[R49] Morello-Frosch R, Cushing LJ, Jesdale BM, Schwartz JM, Guo W, Guo T, Wang M, Harwani S, Petropoulou S-S, Duong W, Park J-S, Petreas M, Gajek R, Alvaran J, She J, Dobraca D, Das R, Woodruff TJ, 2016. Environmental Chemicals in an Urban Population of Pregnant Women and Their Newborns from San Francisco. Environ. Sci. Technol 50 (22), 12464–12472. 27700069 10.1021/acs.est.6b03492 PMC6681912

[R50] Omoike OE, Pack RP, Mamudu HM, Liu Y, Strasser S, Zheng S, Okoro J, Wang L, 2021. Association between per and polyfluoroalkyl substances and markers of inflammation and oxidative stress. Environ. Res 196 10.1016/j.envres.2020.110361. 33131681

[R51] Padula AM, Monk C, Brennan PA, Borders A, Barrett ES, McEvoy CT, Foss S, Desai P, Alshawabkeh A, Wurth R, Salafia C, Fichorova R, Varshavsky J, Kress A, Woodruff TJ, Morello-Frosch R, 2020. A review of maternal prenatal exposures to environmental chemicals and psychosocial stressors-implications for research on perinatal outcomes in the ECHO program. J Perinatol. 40 (1), 10–24. 31616048 10.1038/s41372-019-0510-y PMC6957228

[R52] Panagopoulos Abrahamsson D, Wang A, Jiang T, Wang M, Siddharth A, Morello-Frosch R, Park J-S, Sirota M, Woodruff TJ, 2021. A Comprehensive Non-targeted Analysis Study of the Prenatal Exposome. Environ Sci Technol. 55 (15), 10542–10557. 34260856 10.1021/acs.est.1c01010 PMC8338910

[R53] Radloff LS, 1977. The CES-D Scale: A Self-Report Depression Scale for Research in the General Population. Appl. Psychol. Meas 1 (3), 385–401. 10.1177/014662167700100306.

[R54] Rokoff LB, Rifas-Shiman SL, Coull BA, Cardenas A, Calafat AM, Ye X, Gryparis A, Schwartz J, Sagiv SK, Gold DR, Oken E, Fleisch AF, 2018. Cumulative exposure to environmental pollutants during early pregnancy and reduced fetal growth: the Project Viva cohort. Environ Health. 17 (1) 10.1186/s12940-018-0363-4. PMC5819079 29458383

[R55] Ruiz RJ, Newman M, Suchting R, Pasillas RM, Records K, Stowe RP, Moore TA, 2021. Pregnant Mexican American Biopsychosocial/Cultural risks for adverse infant outcomes. Nursing Open. 8 (2), 709–720. 33570300 10.1002/nop2.676 PMC7877225

[R56] Shrier I, Platt RW, 2008. Reducing bias through directed acyclic graphs. BMC Med. Res. Method 8 (1), 70. 10.1186/1471-2288-8-70. PMC2601045 18973665

[R57] Shu H, Lindh CH, Wikström S, Bornehag CG, 2018. Temporal trends and predictors of perfluoroalkyl substances serum levels in Swedish pregnant women in the SELMA study. PLoS ONE 13 (12). 10.1371/journal.pone.0209255. PMC6312341 30596681

[R58] Steenland K, Barry V, Savitz D. Serum Perfluorooctanoic Acid and Birthweight: An Updated Meta-analysis With Bias Analysis. Epidemiology. 2018;29(6). https://journals.lww.com/epidem/Fulltext/2018/11000/Serum_Perfluorooctanoic_Acid_and_Birthweight__An.4.aspx. 10.1097/EDE.0000000000000903 30063543

[R59] Sunderland EM, Hu XC, Dassuncao C, Tokranov AK, Wagner CC, Allen JG, 2019. A review of the pathways of human exposure to poly- and perfluoroalkyl substances (PFASs) and present understanding of health effects. J Expo Sci Environ Epidemiol. 29 (2), 131–147. 10.1038/s41370-018-0094-1. 30470793 PMC6380916

[R60] Svensson K, Tanner E, Gennings C, Lindh C, Kiviranta H, Wikström S, Bornehag C-G, 2021. Prenatal exposures to mixtures of endocrine disrupting chemicals and children’s weight trajectory up to age 5.5 in the SELMA study. Sci Rep. 11 (1) 10.1038/s41598-021-89846-5. PMC8155069 34040006

[R61] Szegda K, Bertone-Johnson ER, Pekow P, Powers S, Markenson G, Dole N, Chasan-Taber L, 2018. Prenatal Perceived Stress and Adverse Birth Outcomes Among Puerto Rican Women. J Womens Health (Larchmt). 27 (5), 699–708. 29215314 10.1089/jwh.2016.6118 PMC5962329

[R62] Talge NM, Mudd LM, Sikorskii A, Basso O, 2014. United States birth weight reference corrected for implausible gestational age estimates. Pediatrics 133 (5), 844–853. 10.1542/peds.2013-3285. 24777216

[R63] Trowbridge J, Gerona RR, Lin T, Rudel RA, Bessonneau V, Buren H, Morello-Frosch R, 2020. Exposure to Perfluoroalkyl Substances in a Cohort of Women Firefighters and Office Workers in San Francisco. Environ Sci Technol. 54 (6), 3363–3374. 32100527 10.1021/acs.est.9b05490 PMC7244264

[R64] Vesterinen HM, Morello-Frosch R, Sen S, Zeise L, Woodruff TJ, Meliker J, 2017. Cumulative effects of prenatal-exposure to exogenous chemicals and psychosocial stress on fetal growth: Systematic-review of the human and animal evidence. PLoS ONE 12 (7). 10.1371/journal.pone.0176331. PMC5507491 28700705

[R65] Wang A, Abrahamsson DP, Jiang T, Wang M, Morello-Frosch R, Park J-S, Sirota M, Woodruff TJ, 2021. Suspect Screening, Prioritization, and Confirmation of Environmental Chemicals in Maternal-Newborn Pairs from San Francisco. Environ Sci Technol. 55 (8), 5037–5049. 33726493 10.1021/acs.est.0c05984 PMC8114949

[R66] Wolf CJ, Fenton SE, Schmid JE, Calafat AM, Kuklenyik Z, Bryant XA, Thibodeaux J, Das KP, White SS, Lau CS, Abbott BD, 2007. Developmental Toxicity of Perfluorooctanoic Acid in the CD-1 Mouse after Cross-Foster and Restricted Gestational Exposures. Toxicol. Sci 95 (2), 462–473. 17098816 10.1093/toxsci/kfl159

[R67] Wolf CJ, Zehr RD, Schmid JE, Lau C, Abbott BD, 2010. Developmental Effects of Perfluorononanoic Acid in the Mouse Are Dependent on Peroxisome Proliferator-Activated Receptor-Alpha. Corton J, ed. PPAR Research. 2010, 1–11. 10.1155/2010/282896 PMC2948904 20936102

[R68] Xu C, Xu J, Zhang X, Xu S, Liu Q, Weng Z, Gu A, 2021. Serum nickel is associated with craniosynostosis risk: Evidence from humans and mice. Environ. Int 146 10.1016/j.envint.2020.106289. 33276314

[R69] Yao Q, Gao Y, Zhang Y, Qin K, Liew Z, Tian Y, 2021. Associations of paternal and maternal per- and polyfluoroalkyl substances exposure with cord serum reproductive hormones, placental steroidogenic enzyme and birth weight. Chemosphere 285, 131521. 10.1016/j.chemosphere.2021.131521. 34273704

[R70] Zhu Y, Bartell SM, 2020. Per- and polyfluoroalkyl substances in drinking water and birthweight in the US: A county-level study. Environ Epidemiol. 4 (4) 10.1097/EE9.0000000000000107. PMC7595209 33154987

[R71] Zilversmit Pao L, Harville EW, Wickliffe JK, Shankar A, Buekens P, 2019. The Cumulative Risk of Chemical and Nonchemical Exposures on Birth Outcomes in Healthy Women: The Fetal Growth Study. Int J Environ Res Public Health. 16 (19), 3700. 10.3390/ijerph16193700. PMC6801557 31581440

