# Supplementary material for: Prenatal PFAS and psychosocial stress exposures in relation to fetal growth in two pregnancy cohorts: Applying environmental mixture methods to chemical and non-chemical stressors
Source: Environ Int. Author manuscript; Available in PMC 2022 Jun 16. (PMC9202828; doi:10.1016/j.envint.2022.107238)
Supplement: Supplementary Material [file NIHMS1814871-supplement-Supplementary_Material.docx]

Table S1. Method detection limits for PFAS measurements.

| **PFAS Chemical** | **MDL (ng/mL)** |
| --- | --- |
| PFOA | 0.61 |
| PFOSA | 0.014 |
| Me-PFOSA-AcOH | 0.011 |
| Et-PFOSA-AcOH | 0.011 |
| PFUdA | 0.028 |
| PFHxS | 0.018 |
| PFOS | 0.062 |
| PFBS | 0.03 |
| PFNA | 0.042 |
| PFHpA | 0.026 |
| PFDeA | 0.056 |
| PFDoA | 0.061 |

Abbreviations: MDL, method detection limit.

Figure S1. Directed Acyclic Graph (DAG) for the association between PFAS, response to psychosocial stress, and birthweight z-scores.


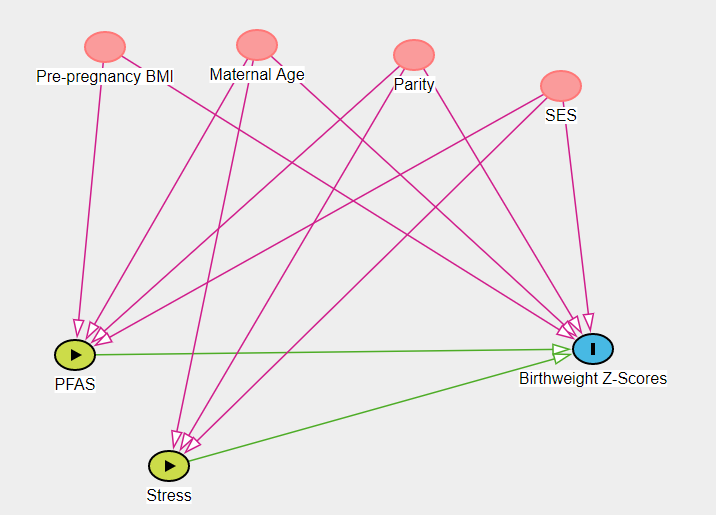


Note: Green indicates exposure, blue indicates outcome and covariates associated with outcome, red indicates covariate associated with both exposure and outcome.

Figure S2. Spearman correlations between natural log transformed per- and poly-fluoroalkyl substances (ng/mL), responses to psychosocial stress (perceived stress and depression), and birthweight for gestational age z-scores (N=876).


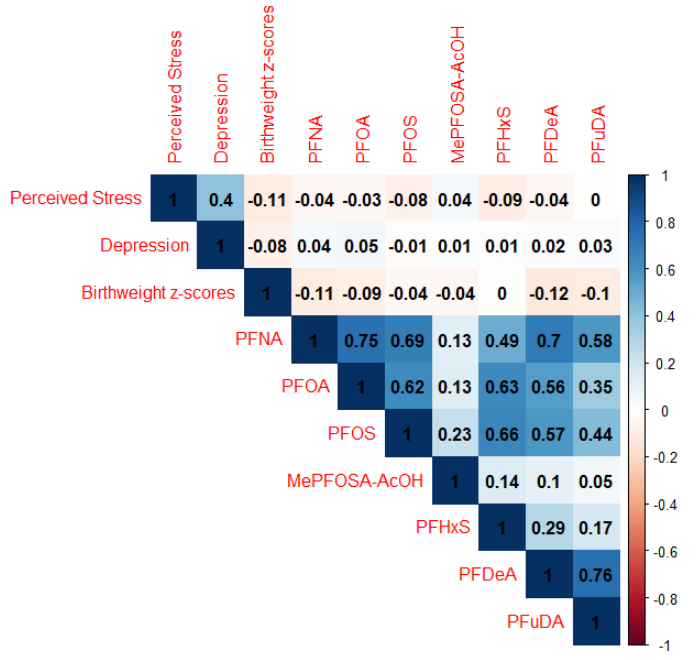


Table S2. Unadjusted and adjusted linear regression coefficients and 95% confidence intervals for association between birthweight z-scores with a one-half interquartile range increase in second trimester PFAS (ng/mL).

|  | **Unadjusted** | | **Adjusted^1^** | |
| --- | --- | --- | --- | --- |
|  | **N** | **β (95% CI)** | **N** | **β (95% CI)** |
| PFNA | 1049 | -0.03 (-0.07, 0) | 964 | -0.01 (-0.05, 0.03) |
| PFOA | 1049 | -0.03 (-0.07, 0.01) | 964 | -0.02 (-0.06, 0.03) |
| PFOS | 1049 | 0 (-0.04, 0.03) | 964 | 0 (-0.03, 0.04) |
| PFHxS | 1049 | 0 (-0.03, 0.04) | 964 | 0 (-0.04, 0.05) |
| Me-PFOSA-AcOH | 1049 | 0 (-0.05, 0.04) | 964 | 0 (-0.04, 0.05) |
| PFDeA | 1049 | -0.04 (-0.08, 0) | 964 | -0.02 (-0.06, 0.03) |
| PFUdA | 1049 | -0.04 (-0.08, -0.01) | 964 | -0.01 (-0.06, 0.03) |

^1^Adjusted for maternal education, age, race/ethnicity, pre-pregnancy BMI, parity, and cohort.

Abbreviations: CI, confidence interval.

Table S3. Unadjusted and adjusted linear regression coefficients and 95% confidence intervals for association between birthweight z-scores with a one-half interquartile range increase measures of psychosocial stress response.

|  | **Unadjusted** | | **Adjusted^1^** | |
| --- | --- | --- | --- | --- |
|  | **N** | **β (95% CI)** | **N** | **β (95% CI)** |
| Perceived Stress T-Score | 1007 | -0.06 (-0.1, -0.02) | 938 | -0.02 (-0.06, 0.03) |
| Depression T-Score | 979 | -0.04 (-0.08, 0) | 900 | -0.03 (-0.07, 0) |

^1^Adjusted for education, age, race/ethnicity, BMI, parity, and cohort.

Abbreviations: CI, confidence interval.

Table S4. Adjusted linear regression coefficients and 95% confidence intervals for the associations between tertiles of PFAS (ng/mL) in maternal serum, tertiles of psychosocial stress response, and birthweight z-scores.

|  | **Unadjusted** | | **Adjusted^1^** | |
| --- | --- | --- | --- | --- |
|  | **N** | **β (95% CI)** | **N** | **β (95% CI)** |
| PFNA |  |  |  |  |
| <0.21 ng/mL | 352 | Ref | 318 | Ref |
| 0.21-0.37 ng/mL | 351 | -0.11 (-0.26, 0.03) | 319 | -0.08 (-0.23, 0.07) |
| >0.37 ng/mL | 346 | -0.18 (-0.33, -0.04) | 327 | -0.11 (-0.26, 0.05) |
| PFOA |  |  |  |  |
| <0.51 ng/mL | 351 | Ref | 312 | Ref |
| 0.51-0.96 ng/mL | 348 | 0.01 (-0.13, 0.16) | 323 | 0.06 (-0.09, 0.21) |
| >0.96 ng/mL | 350 | -0.13 (-0.28, 0.02) | 329 | -0.03 (-0.19, 0.14) |
| PFOS |  |  |  |  |
| <1.41 ng/mL | 354 | Ref | 310 | Ref |
| 1.41-2.59 ng/mL | 345 | 0 (-0.15, 0.14) | 319 | -0.02 (-0.17, 0.13) |
| >2.59 ng/mL | 350 | -0.06 (-0.21, 0.08) | 335 | -0.02 (-0.18, 0.13) |
| PFHxS |  |  |  |  |
| <0.26 ng/mL | 352 | Ref | 308 | Ref |
| 0.26-0.59 ng/mL | 348 | 0.04 (-0.11, 0.18) | 329 | 0.07 (-0.08, 0.23) |
| >0.59 ng/mL | 349 | -0.01 (-0.16, 0.13) | 327 | 0.04 (-0.13, 0.21) |
| Me-PFOSA-AcOH |  |  |  |  |
| <0.03 ng/mL | 351 | Ref | 313 | Ref |
| 0.03-0.06 ng/mL | 348 | -0.27 (-0.41, -0.12) | 323 | -0.17 (-0.32, -0.03) |
| >0.06 ng/mL | 350 | -0.04 (-0.19, 0.1) | 328 | 0.03 (-0.11, 0.18) |
| PFDeA |  |  |  |  |
| <0.07 ng/mL | 350 | Ref | 315 | Ref |
| 0.07-0.14 ng/mL | 351 | -0.07 (-0.21, 0.08) | 328 | -0.07 (-0.22, 0.07) |
| >0.14 ng/mL | 348 | -0.24 (-0.39, -0.09) | 321 | -0.16 (-0.32, 0) |
| PFUdA |  |  |  |  |
| <0.04 ng/mL | 350 | Ref | 313 | Ref |
| 0.04-0.11 ng/mL | 351 | -0.13 (-0.27, 0.02) | 331 | -0.08 (-0.23, 0.06) |
| >0.11 ng/mL | 348 | -0.14 (-0.29, 0) | 320 | 0 (-0.17, 0.18) |
| Perceived Stress T-Score |  |  |  |  |
| Low | 345 | Ref | 331 | Ref |
| Medium | 381 | -0.09 (-0.24, 0.05) | 353 | -0.02 (-0.17, 0.12) |
| High | 281 | -0.16 (-0.32, -0.01) | 254 | -0.03 (-0.19, 0.13) |
| Depression T-Score |  |  |  |  |
| Low | 331 | Ref | 305 | Ref |
| Medium | 368 | -0.08 (-0.22, 0.07) | 332 | -0.02 (-0.17, 0.13) |
| High | 280 | -0.17 (-0.33, -0.01) | 263 | -0.1 (-0.26, 0.05) |

^1^Adjusted for education, age, race/ethnicity, BMI, parity, and cohort.

Abbreviations: CI, confidence interval.

Figure S3. Weights representing the proportion of the positive and negative effects in the (a) overall mixture of PFAS and psychosocial stressors, (b) PFAS and (c) psychosocial stressors in relation to birthweight z-scores (N=876).

Note: Models adjusted for maternal education, age, race/ethnicity, pre-pregnancy BMI, parity, and cohort.

1. Overall B) PFAS


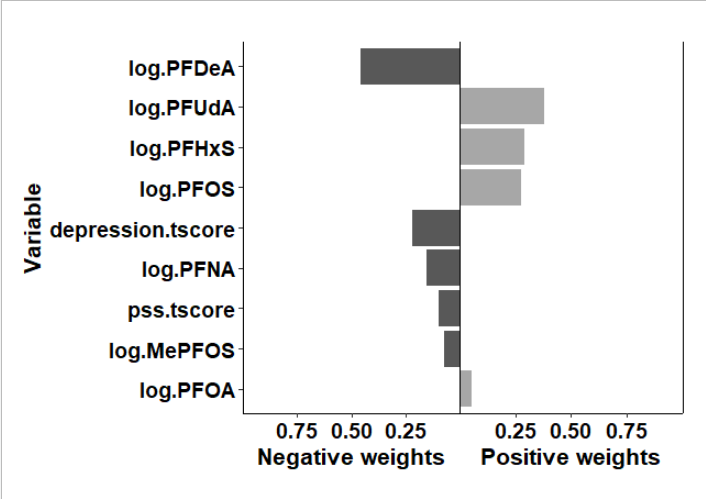

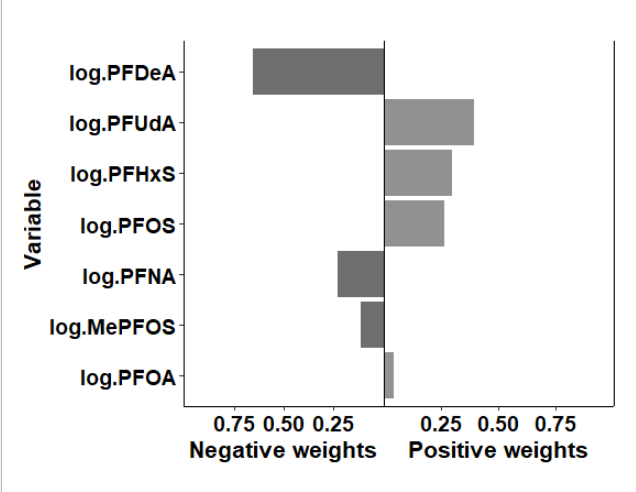


1. Stress


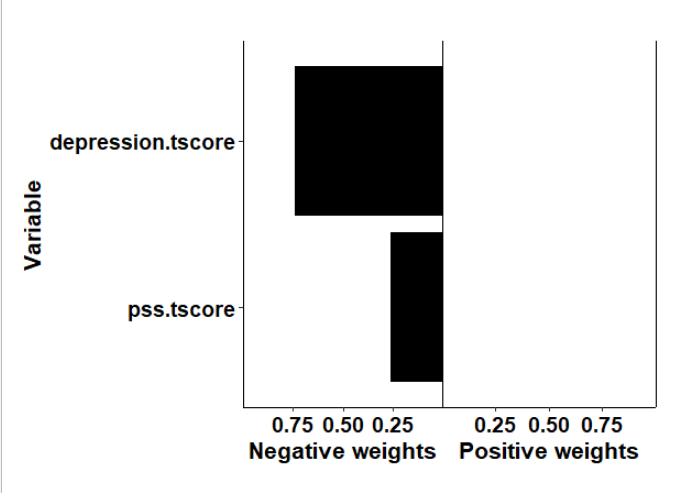


Table S5. Quantile g-computation estimates and 95% confidence interval for the change in birthweight z-scores for a one quartile increase the mixture of PFAS and perceived stress t-scores, removing depression t-scores (N=876).

|  | **β** | **(95% CI)** |
| --- | --- | --- |
| Overall | -0.06 | (-0.18, 0.05) |

Note: Beta estimates are interpreted as the effect on birthweight z-scores of increasing every exposure in the mixture by one quantile. Models are adjusted for maternal education, age, race/ethnicity, pre-pregnancy BMI, parity, and cohort.

Abbreviations: CI, confidence interval.

Table S6. Quantile g-computation estimates and 95% confidence intervals for the change in birthweight z-scores for a one quartile increase the mixture of PFAS and responses to psychosocial stress stratified by cohort.

|  | **β** | **(95% CI)** |
| --- | --- | --- |
| CIOB (N=462) | 0.01 | (-0.18, 0.2) |
| IKIDS (N=414) | -0.11 | (-0.26, 0.05) |

Note: Beta estimates are interpreted as the effect on birthweight z-scores of increasing every exposure in the mixture by one quantile. Models are adjusted for maternal education, age, race/ethnicity, pre-pregnancy BMI, and parity.

Abbreviations: CI, confidence interval.

Figure S4. Weights representing the proportion of the positive and negative effects in the overall mixture of PFAS and psychosocial stressors in relation to birthweight z-scores, stratified by cohort.

Note: Models adjusted for maternal education, age, race/ethnicity, pre-pregnancy BMI, and parity.

1. CIOB (N=462)


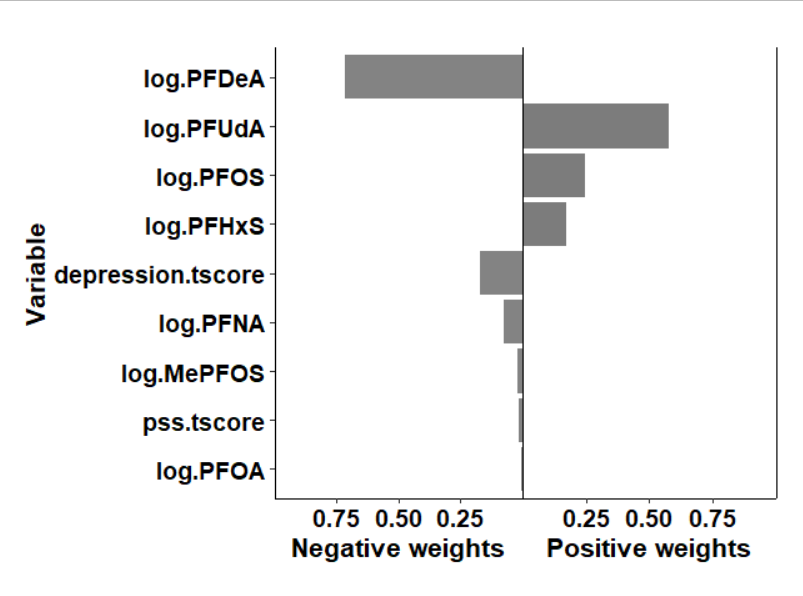


1. IKIDS (N=414)


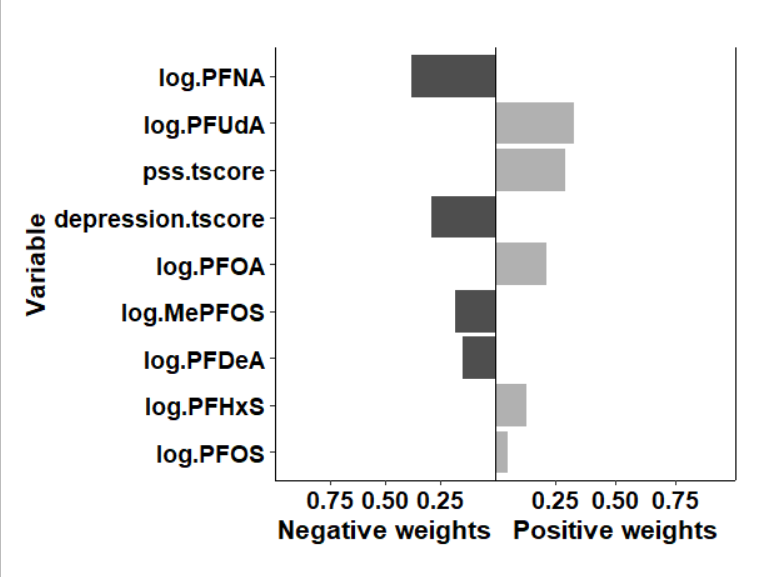


Figure S5. Bivariate exposure–response functions and 95% confidence intervals for the effect of individual PFAS or response to psychosocial stress on birthweight z-scores, conditional on four different quantiles of a second exposure, while fixing the rest of the exposures in the mixture at their 50th percentiles, estimated using BKMR (N=876).


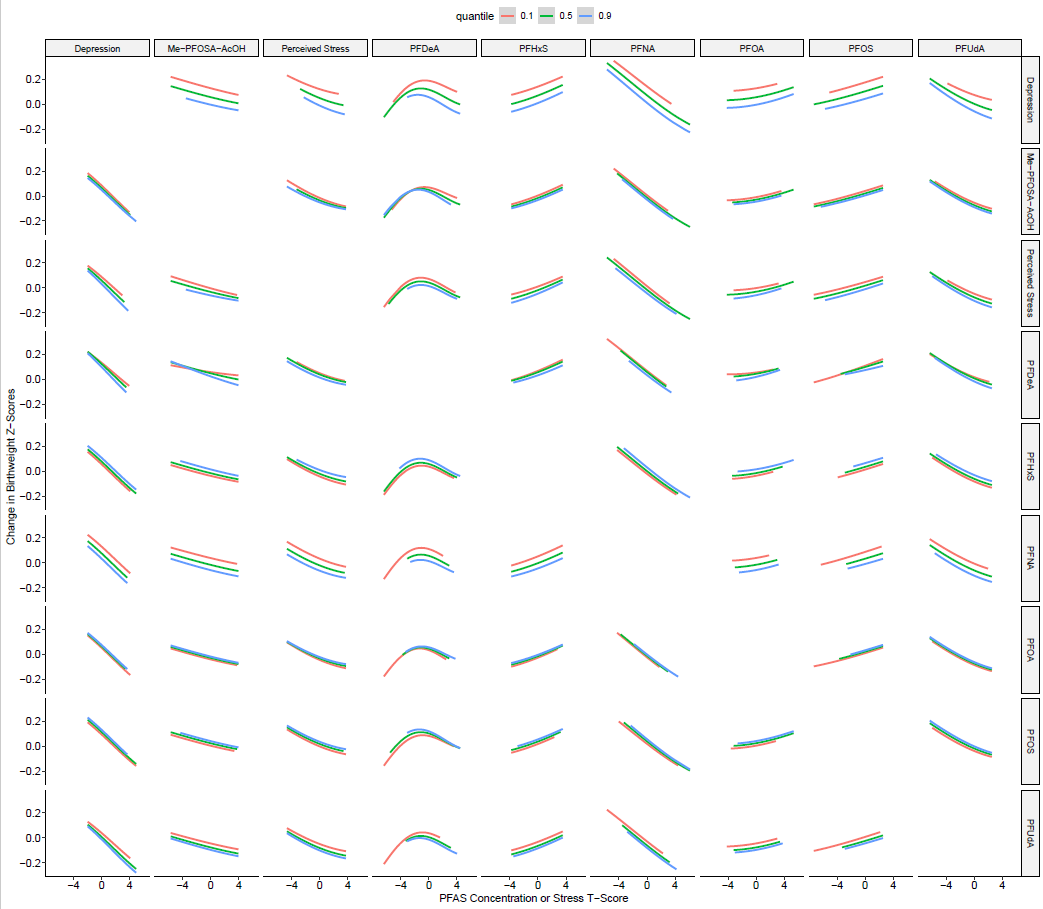


Note: There is evidence of interaction when lines are not overlapping. PFAS were log transformed. All PFAS and responses to psychosocial stress were scaled to have a mean of 0 and standard deviation of 1. Models are adjusted for maternal education, age, race/ethnicity, pre-pregnancy BMI, parity, and cohort.

Table S7. Posterior inclusion probabilities for conditional inclusion into birthweight z-scores measurement model, estimated using Bayesian kernel machine regression (BKMR) (N=876).

|  | Conditional PIP |
| --- | --- |
| PFNA | 0.08 |
| PFOA | 0.05 |
| PFOS | 0.05 |
| PFHxS | 0.05 |
| Me-PFOSA-AcOH | 0.04 |
| PFDeA | 0.15 |
| PFUdA | 0.08 |
| Perceived Stress T-Score | 0.17 |
| Depression T-Score | 0.09 |

Note: PFAS, perceived stress, and depression are scaled to have a mean of 0 and standard deviation of 1. PIP values greater than 0.5 are considered to be important factors.

Abbreviations: PIP, Posterior inclusion probabilities. Models are adjusted for education, age, race/ethnicity, pre-pregnancy BMI, parity, and cohort.

Table S8. Posterior inclusion probabilities for conditional inclusion into birthweight z-scores measurement model stratified by cohort, estimated using Bayesian kernel machine regression (BKMR) (N=876).

|  | IKIDS | CIOB |
| --- | --- | --- |
|  | Conditional PIP | Conditional PIP |
| PFNA | 0.40 | 0.49 |
| PFOA | 0.35 | 0.49 |
| PFOS | 0.31 | 0.43 |
| PFHxS | 0.33 | 0.52 |
| Me-PFOSA-AcOH | 0.34 | 0.51 |
| PFDeA | 0.45 | 0.50 |
| PFUdA | 0.37 | 0.42 |
| Perceived Stress T-Score | 0.41 | 0.51 |
| Depression T-Score | 0.36 | 0.57 |

Note: PFAS, perceived stress, and depression are scaled to have a mean of 0 and standard deviation of 1. PIP values greater than 0.5 are considered to be important factors.

Abbreviations: PIP, Posterior inclusion probabilities. Models are adjusted for education, age, race/ethnicity, pre-pregnancy BMI, and parity.

Figure S6. Cumulative effect (estimates and 95% credible intervals) of the PFAS and perceived stress t-scores mixture on birthweight z-scores, removing depression t-scores, estimated using BKMR (N=876).


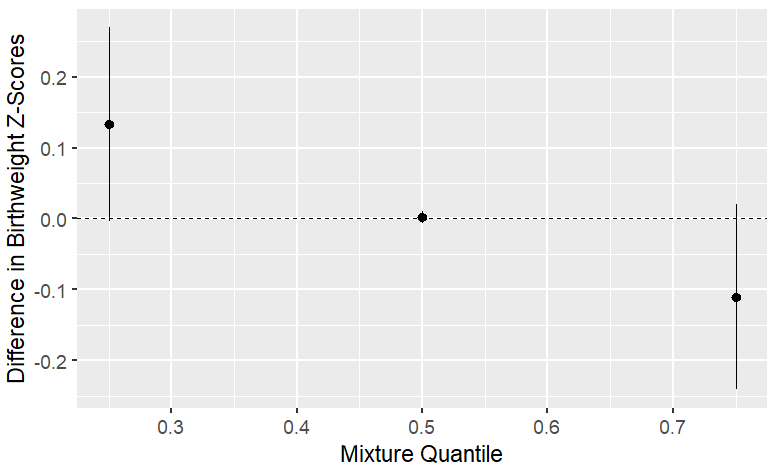


Note: PFAS were log trans formed. All PFAS and responses to psychosocial stress were scaled to have a mean of 0 and standard deviation of 1. Models are adjusted for maternal education, age, race/ethnicity, pre-pregnancy BMI, parity, and cohort.

Figure S7. Cumulative effect (estimates and 95% credible intervals) of the PFAS and response to psychosocial stress mixture on birthweight z-scores stratified by cohort, estimated using BKMR.


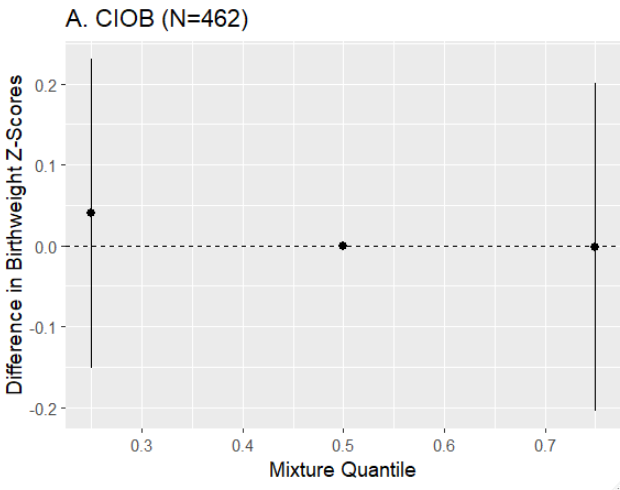


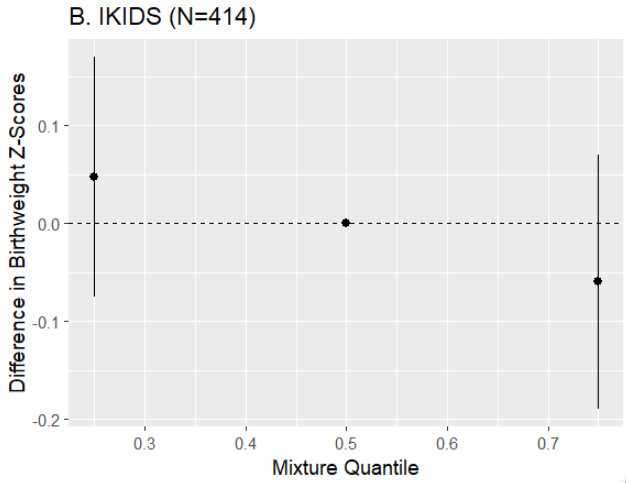


Note: PFAS were log transformed. All PFAS and responses to psychosocial stress were scaled to have a mean of 0 and standard deviation of 1. Models are adjusted for maternal education, age, race/ethnicity, pre-pregnancy BMI, and parity.
